# Discovery of Boolean metabolic networks: integer linear programming based approach

**DOI:** 10.1186/s12918-018-0528-3

**Published:** 2018-04-11

**Authors:** Yushan Qiu, Hao Jiang, Wai-Ki Ching, Xiaoqing Cheng

**Affiliations:** 1 College of Mathematics and Statistics, Shenzhen University, Nanhai Avenue 3688, Shenzhen, 518060 China; 2 Department of Mathematics, School of Information, Renmin University of China, No.59 Zhong Guan Cun Avenue, Hai Dian District, Beijing, 100872 China; 3 Department of Mathematics, The University of Hong Kong, Pokfulam Road, Hong Kong, Hong Kong; 4 School of Mathematics and Statistics, Xi’An Jiaotong University, No.28 West Xianning Road, Xi’An, 710049 China

**Keywords:** Metabolic network, Integer linear programming, Boolean model

## Abstract

**Background:**

Traditional drug discovery methods focused on the efficacy of drugs rather than their toxicity. However, toxicity and/or lack of efficacy are produced when unintended targets are affected in metabolic networks. Thus, identification of biological targets which can be manipulated to produce the desired effect with minimum side-effects has become an important and challenging topic. Efficient computational methods are required to identify the drug targets while incurring minimal side-effects.

**Results:**

In this paper, we propose a graph-based computational damage model that summarizes the impact of enzymes on compounds in metabolic networks. An efficient method based on Integer Linear Programming formalism is then developed to identify the optimal enzyme-combination so as to minimize the side-effects. The identified target enzymes for known successful drugs are then verified by comparing the results with those in the existing literature.

**Conclusions:**

Side-effects reduction plays a crucial role in the study of drug development. A graph-based computational damage model is proposed and the theoretical analysis states the captured problem is NP-completeness. The proposed approaches can therefore contribute to the discovery of drug targets. Our developed software is available at “http://hkumath.hku.hk/~wkc/APBC2018-metabolic-network.zip”.

## Background

One of the most important biological processes in organism is metabolism and its dysregulation can contribute to many human diseases. The manipulation of metabolism has been extensively studied in the field of metabolic engineering as many of metabolic process produce commodity and specialty chemicals. In particular, production of industrially valuable products using a microbial host with recombinant technology becomes one of the most successful applications of metabolic engineering [1–3]. Metabolic network is used to model the behavior of reactions and chemicals [4]. Authors in [5] have proposed a new mathematical model for large metabolic network and demonstrated that the outcome of network expansion is robust against of single or few reactions. Indeed, for the analysis of metabolic networks, many studies have been conducted so as to develop Boolean models. For example, Smart et al. [6] studied the effect of deletion of each enzyme in metabolic network of a Boolean model, and Lemke et al. [7] considered almost the same problem from the viewpoint of the Boolean aspect. Tamura et al. [8] developed an integer linear programming method for Boolean reaction cut (BRC) problem. Furthermore, in [9], the computational complexity of BRC was analyzed. In the Boolean model of metabolic networks, compounds and reactions can be represented by “OR” and “AND” nodes, respectively. Li et al. [10] have developed methods for finding a set of enzymes whose inhibition stops the production of the target compound with a minimum elimination of non-target compounds. Furthermore, Takemoto et al. [11] and Lee et al. [12] estimated the size distribution of the deletion effect of enzymes using a branching process. Enumerating all the possible enzyme combination is infeasible since the number of combination increases exponentially with the number of enzymes. Lu et al. [13] developed an integer linear programming method for designing synthetic metabolic networks by considering minimum reaction insertion in a Boolean model. The proposed method can appropriately solve minimum reaction insertion in a Boolean network.

In pharmaceutics, the development of drugs mainly focus on target identification and lead inhibitor identification [14]. Furthermore, many researchers have done a lot of work on the efficacy of drugs regardless of their side-effects (toxicity). However, toxicity or lack of efficacy may occur when the compounds which are not the intended targets in the metabolic network. Thus how to effectively reduce the side-effect of drugs has become an important and challenging issue. The current works aim to identify the biological targets (could be enzyme or protein) for drugs which can be manipulated so as to produce the desired effect (i.e., curing a disease) with minimum disruptive side-effects [15, 16]. Drug targets, in particular enzymes, are chosen to reduce abnormal metabolites by formulating an optimal combination problem in enzyme combination in metabolic networks [10, 17]. It is known that the function of enzymes is to catalyze reactions and produces metabolites (compounds) in metabolic networks. Human diseases, especially metabolic diseases, may be directly caused by the accumulation of certain compounds due to the malfunctions of enzymes. We denote such compounds as *target compounds* while the others as *non-target compounds*. For example, the malfunction of enzyme phenylalanine hydroxylase results in accumulation of the amino acid phenylalanine which causes the disease phenylketonuria [18]. Therefore, there is a great need to identify a set of enzymes which are manipulated by drugs to prevent the buildup of target compounds with minimal damage. Here *damage* corresponds to the number of non-target compounds whose production are forced to stop by the inhibition of that enzyme (or enzyme set).

Then the problem becomes to identify the optimal enzyme set whose inhibition eliminates the target compounds and at the same time, incurs minimum damage based on the given metabolic network and a set of target compounds. We denote such problem as enzyme combination identification (ECI). Sridhar et al. [19] developed a branch-and-bound algorithm to dynamically explore the search space. Furthermore, two filtering strategies are proposed to prune the search space to guarantee an optimal solution. However, their algorithm is complicated and impractical for us to use it. In this paper, we develop an efficient method based on Integer Linear Programming [20–22] which has a wide application in solving NP-hard problems. Furthermore, all instances of the original problem are needed to convert into integer programming formalization so as to apply the existing free solver called CPLEX [23].

This paper has two main contributions. First, we formulate a new biological problem based on Boolean metabolic network to identify the drug target with minimum damage. Second, we propose an effective and efficient method, Integer Linear Programming, (needs *O*(*m*+*n*) variables) to solve the captured problem and it is easy to implement. By integrating the human metabolic networks, we have shown that the proposed approach can accurately identify the target enzyme set for known drugs in the literature. Furthermore, experiments have shown that our proposed method is extremely efficient which can effectively solve the problem in seconds.

The remainder of the paper is organized as follows. In “Problem formulation” section, we formulate the captured problem. “Methods” section presents the algorithm ILP-ECI. In “Results and discussions” section, we discuss the experimental results and give a theoretical illustration. Finally, conclusions are given in the last section.

## Problem formulation

A metabolic network can be represented by a directed graph *G*=(*V,A*) which captures the interaction between reactions, compound, and enzymes. Let *C*, *R*, and *E* denote the set of compounds, reactions, and enzymes, respectively. Then *V*_*C*_,*V*_*R*_ and *V*_*E*_ denote the set of vertices from *C*, *R*, and *E*, where $\phantom {\dot {i}\!}V_{C}=\{v_{c_{1}},v_{c_{2}},\cdots,v_{c_{m}}\}$ denotes a set of *compound nodes*, $\phantom {\dot {i}\!}V_{R}=\{v_{r_{1}},v_{r_{2}},\cdots,v_{r_{n}}\}$ represents a set of *reaction nodes* and $\phantom {\dot {i}\!}V_{E}=\{v_{e_{1}},v_{e_{2}},\cdots,v_{e_{l}}\}$ defines a set of *enzyme nodes*. The subscript *m,n,l* denote the number of *compound nodes*, *reaction nodes* and *enzyme nodes*, respectively. Here *V*=*V*_*C*_∪*V*_*R*_∪*V*_*E*_ and *V*_*C*_∩*V*_*R*_={},*V*_*C*_∩*V*_*E*_={},*V*_*R*_∩*V*_*E*_={} hold. Here {} is the empty set. Let *V*_*s*_⊆*V*_*C*_ and *V*_*t*_⊆*V*_*C*_ be sets of *source nodes* and *target nodes*, respectively. Source nodes are those nodes having no incoming edges and they are the seed compounds. A metabolic network can be defined as follows:

- For each edge (*u,v*)∈*A*, either (*u*∈*V*_*C*_)∧(*v*∈*V*_*R*_) or (*u*∈*V*_*R*_)∧(*v*∈*V*_*C*_|*v*∈*V*_*E*_) holds and “ ∧” represents “AND” function;
- Each source node has no incoming edges;
- For nodes *v*∉*V*_*s*_ and *v*∈*V*_*C*_ have at least one incoming edge.

The main problem **Enzyme Combination Identification (ECI)** in a Boolean network is first described with a simple example and then followed by its mathematical formalization.

**Fig. 1:**
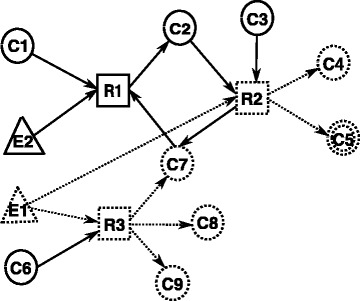
A graph constructed for a hypothetical metabolic network

A small hypothetical metabolic network is shown in Fig. 1, where circles, rectangles and triangles represent compounds, reactions and enzymes, respectively. For instance, *R*_1_ is a reaction, its substrates are *C*_1_ and *E*_2_ and its product is *C*_2_. Here *C*_5_ is the target compound in this figure which shall be stopped. To stop the production of *C*_5_, reaction *R*_2_ must be prevented from taking place. One of the possible solution is to disrupt one of its catalyzing enzymes (*E*_1_ for instance). Another is to stop the production of its reactant compounds (i.e., *C*_2_ or *C*_3_). If *C*_2_ is stopped, then two possible ways can achieve this effect (i.e., enzyme *E*_2_ or *E*_1_). It is noted that *C*_2_,*R*_2_,*C*_7_ and *R*_1_ forms a cycle, the inhibition of *E*_1_ will result in inactivating reaction *R*_2_ which will further makes *C*_2_ inproducible. The other way is inhibiting enzyme *E*_2_ which makes *R*_1_ inactive and further stop the production of compound *C*_2_. Therefore, the inhibition of enzyme *E*_1_ or *E*_2_ can result in stopping the production of the target compound.

We define the **Enzyme Combination Identification** as follows.

- **Input:** A metabolic network and a set of target compounds *T*(*T*∈*C*), *C* is the set of compounds.
- **Output:** Find a set of enzymes *X*(*X*∈*E*) with minimum damage, whose inhibition stops the production of all the compounds in *T*.

For simplicity, we assume there are no external inputs to all reactions and all the input compounds related to reactions are shown in the network. We note that different compounds and enzymes may have different levels of importance in the metabolic networks. Here we assume that all the compounds and enzymes are of equal importance. We assign binary value (i.e., 0 or 1) to each node *V* in the Boolean model. “0” means that the corresponding compound is not producible or the corresponding reaction is inactive while “1” means that the corresponding compound is producible and the corresponding reaction is active. For the related enzyme catalyzing the reaction, “1” means that it is active and “0” means that it is inhibited. Let *G* be such an assignment (*G* is a function from *V* to {0,1}). For each node *v*∈*V*, we write *v*=1 (resp., *v*=0) if 1 (resp., 0) is assigned to *v*. Then *G* can be regarded as a *valid assignment* if the following conditions are satisfied:

- (i) For each *v*∈*V*_*s*_,*v*=1.
- (ii) For each *v*∉*V*_*s*_,*v*=1 if and only if there is *u* such that (*u,v*)∈*E* and *u*=1.
- (iii) For each *v*∈*V*_*r*_,*v*=1 if and only if *u*=1 holds for all *u* such that (*u,v*)∈*E*.

Condition (ii) implies that compound nodes correspond to “OR” nodes. Condition (iii) implies that reaction nodes correspond to “AND” nodes, which means that the output is forced to 0 when a node is inactivated.

From Fig. 1, we can see that inhibition of *E*_1_ results in the knock out of compounds *C*_4_,*C*7,*C*_8_ and *C*_9_ in addition to the target compound *C*_5_. Then we denote the number of non-target compounds knocked out as the *damage*, which is caused by the manipulating the enzyme set in the metabolic network. It can be seen that the damage of inhibiting *E*_2_ is 2 (i.e., *C*_2_ and *C*_4_). Compound *C*_7_ is still producible because it can be produced by *R*_3_ even after the inhibition of *E*_2_. The damage effect of inhibition of *E*_1_ is 4 (i.e., *C*_4_,*C*7,*C*_8_ and *C*_9_). Both *E*_1_ and *E*_2_ are potential drug targets since they can achieve the effect of disrupting the target compound *C*_5_. However, *E*_2_ is a better drug target than *E*_1_ owing to the fact that it causes less damage.

## Methods

In this section, we introduce integer programming-based methods for ECI. Integer programming, in particular, Integer Linear Programming (ILP) is set to minimize (or maximize) a linear objective function under linear constraints with all the variables taking integer values. In the following, each variable takes including the binary value (i.e., 0 or 1), representing the Boolean values. We apply ILP to ECI since ILP is widely used for solving NP-hard problems.

The ILP formulation for the network in Fig. 1 is as follows: **ILP-ECI**

1 $$\begin{array}{*{20}l} {\min} \left\{\sum_{i=1}^{m}TCi\right\} \end{array} $$

subject to

2 $$\begin{array}{*{20}l} &FC5=1  \end{array} $$

3 $$\begin{array}{*{20}l} &TR1+FC1+FC7+FE2\geq 1,  \\ &FR1+TC1\geq 1, \\ &FR1+TC7\geq 1, \\ &FR1+TE2\geq 1, \end{array} $$

4 $$\begin{array}{*{20}l} &TR2+FC2+FC3+FE1\geq 1, \\ &FR2+TC2\geq 1,\\ &FR2+TC3\geq 1,\\ &FR2+TE1\geq 1, \end{array} $$

5 $$\begin{array}{*{20}l} &TR3+FC6+FE1\geq 1, \\ &FR3+TC6\geq 1,\\ &FR3+TE1\geq 1 \end{array} $$

6 $$\begin{array}{*{20}l} &TC2=TR1 \end{array} $$

7 $$\begin{array}{*{20}l} &TC4=TR2 \end{array} $$

8 $$\begin{array}{*{20}l} &TC5=TR2 \end{array} $$

9 $$\begin{array}{*{20}l} &FC7+TR2+TR3\geq 1,\\ &TC7+FR2\geq 1,\\ &TC7+FR3\geq 1 \end{array} $$

10 $$\begin{array}{*{20}l} &TC8=TR3 \end{array} $$

11 $$\begin{array}{*{20}l} &TC9=TR3 \end{array} $$

12 $$\begin{array}{*{20}l} &TC1=1, \qquad TC3=1, \qquad TC6=1 \end{array} $$

13 $$\begin{array}{*{20}l} &TX+FX=1 \text{for any X}  \end{array} $$

We denote the above formalization as **ILP-ECI**. Here all variables including the value of reaction compound and enzyme nodes take either 1 or 0. Thus, $v_{r_{i}}$ can be either 0 or 1, and $v_{c_{i}}$ and $v_{e_{i}}$ also take 0 or 1. In this example, $v_{r_{i}}=0$ (resp. $v_{r_{i}}=1$) indicating that the value of reaction *i* takes 0 is represented by *FRi*=1 (resp. *TRi*=1) which implies that the reaction is inactivated (otherwise, the *TRi*=1 implies the reaction is activated). Therefore, *TRi*=0 (equivalent to *FRi*=1) means the corresponding value for true reaction takes 0, which implies the reaction is inactive. And *FRi*=0 (equivalent to *TRi*=1) indicates the corresponding value for false reaction takes 0, which implies the reaction is active. Thus, *TRi*+*FRi*=1 holds for any node *i* in the network. Similarly, *TCi* and *FCi* are used to represent the values of compound nodes. For instance, *TC*2=1 means that $v_{c_{2}}=1$ and in other words, *FC*2=0 since *TCi*+*FCi*=1. Furthermore, $v_{r_{i}}$ corresponds to “AND” node which implies that if $v_{e_{i}}=0$ will inactivate $v_{r_{i}}$.

The objective function (1) means that the damage should be minimized. *FCi*=1 (or *TCi*=0) means that a compound $v_{c_{i}}$ is not producible. Equation (2) means that the target compound $v_{c_{5}}$ should be 0 after the 0-1 assignment converges. Equation (3) represents the Boolean relation $v_{r_{1}}=v_{c_{1}}\land v_{c_{7}}\land v_{e_{2}}$. Note that the Boolean relations such as “ ∨” or “ ∧” cannot be used in ILP formulation, we need to convert them into linear equations and/or inequations. Actually, “ ∨” indicates “AND” function and “ ∧”represents “OR” function. Since *x*_1_=*x*_2_∧*x*_3_∧⋯∧*x*_*n*_ can be represented by

$${}\left(x_{1}\!\vee\! \overline{x_{2}} \vee\! \cdots \!\vee \overline {x_{n}}\right)\land\left(\overline{x_{1}} \vee x_{2}\right)\land \left(\overline{x_{1}} \vee x_{3}\right)\land\! \cdots \land \left(\overline{x_{1}} \vee\! x_{n}\right)\,=\,1, $$

the constraint $v_{r_{1}}=v_{c_{1}}\land v_{c_{7}}\land v_{e_{2}}$ can be converted into

$${}\left(v_{r_{1}}\!\vee \!\overline{v_{c_{1}}}\!\vee\! \overline{v_{c_{7}}}\vee \overline{v_{e_{2}}}\right)\land \left(\overline{v_{r_{1}}}\vee \!v_{c_{1}}\right)\land \left(\overline{v_{r_{1}}}\vee\! v_{c_{7}}\right)\land\left(\overline{v_{r_{1}}}\!\vee \!v_{e_{2}}\right)\,=\,1. $$

Thus Eq. (3) is obtained. Similarly, Eqs. (4)-(5) represent the constraints of $v_{r_{2}}$ and $v_{r_{3}}$, respectively.

For a compound node with indegree is 1 which indicates the node has only one incoming edge, the value of the predecessor is just copied. For instance, since $v_{c_{2}}$ has only one predecessor $v_{r_{1}}$, $v_{c_{2}}$ is just copied from $v_{r_{1}}$ as shown in Eq. (6). Similarly, $v_{c_{4}}$ is just copied from $v_{r_{2}}$ which is shown in Eq. (7).

However, for a compound node with indegree more than 1, it is necessary to convert the “ ∨” relation into linear equation or inequations. Equation (9) represents the Boolean relation $v_{c_{7}}=v_{r_{2}}\vee v_{r_{3}}$. Since *x*_1_=*x*_2_∨*x*_3_∨⋯∨*x*_*n*_ can be represented by

$${}\left(\overline{x_{1}}\!\vee\! {x_{2}} \!\vee\! \cdots \!\vee\! { x_{n}}\right)\land\left({x_{1}} \vee \overline{x_{2}}\right)\land \left({x_{1}} \vee \overline{x_{3}}\right)\land \cdots \land \left(x_{1} \!\vee \overline{x_{n}}\right)\,=\,1, $$

$v_{c_{7}}=v_{r_{2}}\vee v_{r_{3}}$ can be turned into $\left (\overline {v_{c_{7}}}\vee v_{r_{2}}\vee v_{r_{3}}\right)\land (v_{c_{7}}\vee \overline {v_{r_{2}}})\land \left (v_{c_{7}}\vee \overline {v_{r_{3}}}\right)$. Thus, Eq. (9) can be obtained.

Equation (6) - Eq. (12) represent the constraints of $v_{c_{1}},\cdots,v_{c_{9}}$ respectively. Equation (12) means that $v_{c_{1}}, v_{c_{3}}$ and $v_{c_{6}}$ are 1 since their indegrees is 0.

Equation (13) means that “T” and “F” correspond to “true (1)” and “false (0)”, respectively, and complement each other. *X* in Eq. (13) means any component or reaction in the metabolic network.

The above formalization can clearly solve ECI and obtain the correct solution {*E*_2_}. Besides, the number of variables is *O*(*m*+*n*) in the above formalization where *m* and *n* are the number of compounds and reactions, respectively.

It is noted that solving ILP is NP-complete, however, a problem that can be formalized as ILP is not always NP-complete. Thus in the following, we prove that ECI is NP-complete.

### **Theorem**

ECI is NP-complete problem with the maximum indegree and outdegree being bounded by 2.

### *Proof*

**Fig. 2:**
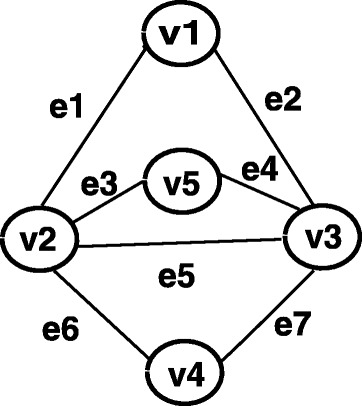
An instance of minimum edge cover (MEC)

Obviously, the problem is in NP, it suffices to show that it is NP-hardness. The proof is by a polynomial time reduction from minimum edge cover (MEC), which is a problem for a given graph to find the minimum number of edges so that each node is incident to at least one of the selected edges. For instance, *E*_1_={*e*_2_,*e*_3_,*e*_6_} is one of optimal solutions of MEC for graph shown in Fig. 2. Let *G*=(*V,E*) be an instance of MEC, where *V*={*v*_1_,*v*_2_,⋯,*v*_*n*_} and *E*={*e*_1_,*e*_2_,⋯,*e*_*m*_}. We then construct the corresponding ECI as below. The metabolite network *G*=(*V*_*c*_∪*V*_*r*_∪*V*_*e*_,*E*) is given by

$$\begin{aligned} V_{c}=\left\{c_{1},c_{2},\cdots,c_{m}\right\}\cup{c_{t}}, \quad V_{r}=\left\{r_{1},r_{2},\cdots,r_{n}\right\}, \end{aligned} $$

$$\begin{aligned} &{}E\,=\,\left\{\!\left\{\!c_{i},r_{j}\right\}\!|i\,=\,1,\cdots, m, j\,=\,\!1,\cdots, n, \text{if} \ v_{j} \ \text{is an end point of}\right.\\ &{}\left. \ e_{i}.\right\} \cup \left\{\left\{r_{j},c_{t}\right\}|j=1,\cdots,n\right\} \end{aligned} $$

**Fig. 3:**
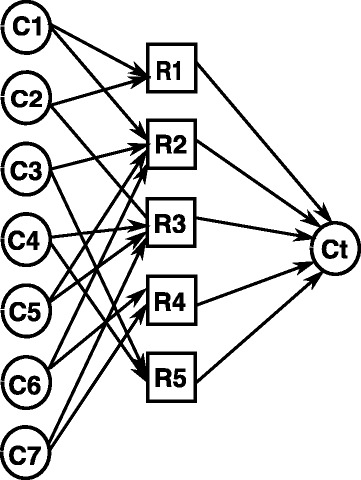
The polynomial time reduction from minimum edge cover (MEC) problem to minimum damage identification problem

It is noted that the minimum *damage* is determined uniquely by the inhibition of enzyme set. Furthermore, our objective is to minimize the “damage” (side-effects). Then *V*_*e*_ can be regarded as virtual nodes and denoted as an empty set in this case. The ECI problem can be converted into the problem of identifying the minimum set of non-target compounds. Thus the graph for MEC shown in Fig. 2 is converted into ECI shown in Fig. 3. It is clear that this conversion can be done in polynomial time. Then we show that MEC for *G* has a solution of size *z* if and only if ECI has a minimum damage of size *z*. To guarantee that the target compound *c*_*t*_ is stopped (i.e., *c*_*t*_=0), it implies that all *r*_*j*_ (*j*=1,⋯,*n*) takes the value 0. If *G* has an edge cover of size *z*, then it follows that the minimum number of *c*_*i*_ taking 0 should be *z*. On the other hand, if the minimum damage for ECI is *z*, then each *r*_*j*_ must be 0 so as to satisfy *c*_*t*_=0, we have at least predecessor of each *r*_*j*_ must be included in the minimum damage set. Since there is an edge between *c*_*i*_ and *r*_*j*_ if and only if *v*_*j*_ is incident to *e*_*i*_. Thus {*c*_*i*_|*c*_*i*_∈minimum damage set} is an edge cover of size *z*. □

## Results and discussions

In this section, we verify the biological validity of our proposed method by using known drugs. Besides, the performance of the ILP-ECI algorithm is evaluated by using the execution time which indicates the total time taken by the method. For the experimental data, we extracted the metabolic network information from the Kyoto Encyclopedia of Genes and Genomes (KEGG) database [24]. KEGG is database which provides known drug molecules along the the enzymes they inhibit and their therapeutic category. Then we use drugs at this database as our benchmarks and we report two of them due to the space limitation. And the value in parenthesis that starts with letter “C”, “D”, and “E” (e.g., E1.13.11.34) is the unique identifier assigned to the corresponding compound, drug, and enzyme respectively in KEGG.

**Fig. 4:**
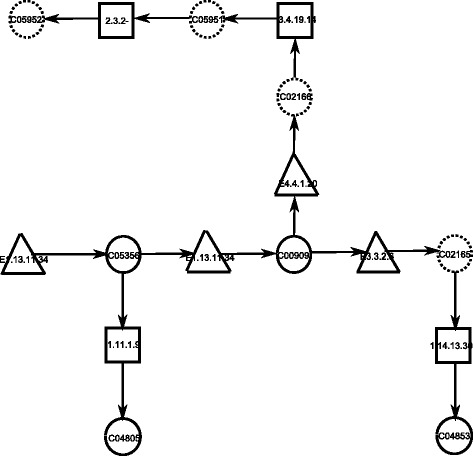
Subgraph of arachidonic acid metabolism

The drug we used in this paper is *Benoxaprofen* (D03080) which inhibits arachidonate 5-lipoxygenase (E1.13.11.34) [25]. This enzyme appears in several networks including arachidonic acid metabolism network (hsa00590). In Pharmacology, the inhibition of 5-lipoxygenase will decrease the biosynthesis of LTB4 (C02165), cysteinylcontaining leukotrienes LTC4 (C02166), LTD4 (C05951) and LTE4 (C05952) (see, for instance, Fig. 4) [26]. According to our graph model, the removal of E1.13.11.34 will eliminate four compounds (LTB4, LTC4, LTD4 and LTE4) in arachidonic acid metabolism network. Furthermore, these four compounds play an important role in the mechanisms of toxic brain damage in acute methanol poisoning in humans [27]. Thus, we chose them as the target compounds. Apart from that, inhibiting this enzyme also eliminates four more compounds (i.e., 5(S)-HPETE(C05356), 5-HETE(C04805), LTA4(C00909), and 20-OH-LTB4(C04853)) [28]. These compounds can be regarded as “damage” in our model. Furthermore, when applying ILP-ECI with LTB4, LTC4, LTD4 and LTE4 as the target compounds, we find LTA4H (E3.3.2.6) and LTC4 synthase (E4.4.1.20) as the optimal enzyme set since the inhibition of these two enzymes eliminates only one non-target compound namely 20-OH-LTB4 (C04853). Thus, it is shown that ILP-ECI potentially finds a better solution in this experiment than the existing drugs since the same target compounds are eliminated by the existing drug in addition to other four compounds. Indeed, recent research validated our model since the anti-inflammatory effect of the levels of LTA4H [29] and LTC4 [30] has been observed. The computational time in this experiment takes only 5.19 s.

Another experiment we conducted is the histidine metabolism network (hsa00340). The enzyme amine oxidase (E.1.4.3.4) appeared in hsa00340 can be inhibited by drug (Rasagiline (D02562)). According to our graph model, the removal of enzyme E.1.4.3.4 will result in eliminating two compounds Methylimidazoleacetic acid (C05828) and Methylimidazole acetaldehyde (C05827). It should be noted that the level of pros-methylimidazoleacetic acid is closely related to severity of Parkinson disease in patients [31, 32]. Running ILP-ECI with Methylimidazoleacetic acid and Methylimidazole acetaldehyde as the target compounds finds amine oxidase as the optimal enzyme. It takes only 3.26 s to run ILP-ECI. This experiment verifies that Rasgiline targets the optimal enzyme. An important advantage of our Boolean model is its capability of detecting the lack of substrates where the connectivity-based methods fail to handle this. Another advantage of this model is its capability of handling branches and cycles in a pathway from the source compound to the target compound. However, there are still have some limitations in this method. One of the major drawbacks is the assumption that all the compounds and enzymes are of equal importance. However, different compounds and enzymes may have different levels of importance in the metabolic networks. Our future work will focus on developing other models which include the weight of different nodes.

## Conclusions

In this paper, we formulate the optimal enzyme combination identification (ECI) problem as an optimization problem in Boolean metabolic networks. We have proven that ECI in the Boolean model is NP-complete and the target enzyme set is uniquely determined when the target compounds are given. Furthermore, considering that an exhaustive search cannot be used to solve ECI when the network is large, we developed an ILP-based algorithm for ECI. Considering that the computational time of IP-based method is exponential to the number of variables, to improve the scalability of the developed method, it is vital to reduce the number of variables appearing in IP formalization. And our proposed IP-based method needs *O*(*m*+*n*) variables.

The efficiency and effectiveness are validated by the computational experiments in which datasets were downloaded from the KEGG database. The results demonstrate that the proposed model can accurately identify the target enzymes for known successful drugs in the literature. Specifically, ILP-ECI has found a different enzyme set for the target compounds of Benoxaprofen which indicates that our method has a great potential to be better than Benoxaprofen. The reason is that the solution of our algorithm damages only one non-target compound while Benxaprofen damages 4 non-target compounds including the compound damaged by ILP-ECI’s solution. Besides, ILP-ECI has found that the same target enzyme like Rasagiline when its target compounds are given in advance. It is to be noted that our problem needs only *O*(*m*+*n*) variables in the IP formalization. The experiments also show that ILP-ECI can solve the problem in a short time which confirms the efficiency of our algorithm.
